# Clinical Analysis of Bloodstream Infection of *Escherichia coli* in Patients with Pancreatic Cancer from 2011 to 2019

**DOI:** 10.1155/2022/1338188

**Published:** 2022-03-16

**Authors:** Changsen Bai, Xiuse Zhang, Dong Yang, Ding Li, Honglei Feng, Yueguo Li

**Affiliations:** Department of Laboratory, National Clinical Research Center for Cancer, Key Laboratory of Cancer Prevention and Therapy, Tianjin's Clinical Research Center for Cancer, Tianjin Medical University Cancer Institute and Hospital, Tianjin, China

## Abstract

**Background:**

Pancreatic cancer patients were particularly predisposed to develop *Escherichia coli (E. coli)* bloodstream infection (BSI); however, little information is currently available. We set out to find *E. coli* BSI's risk factors in pancreatic cancer to provide valuable experience.

**Methods:**

We retrospectively analyzed the clinical data of pancreatic cancer patients (31 cases with *E. coli* BSI and 93 cases without BSI) by a case-control study. SPSS 17.0 was adopted to perform univariate and multivariate analyses. Bacterial resistance analysis was performed by Whonet 5.6.

**Results:**

Hospitalization days ≥7 days, number of admissions ≥2 times, surgery, chemotherapy, the type of antibiotics used ≥2 species, albumin<40.0 g/L, and prealbumin < 0.2 g/L were the potential risk factors for pancreatic cancer patients with *E. coli* BSI (*P* < 0.1). Multivariate logistic regression showed hospitalization days ≥7 days (OR = 11.196, 95% CI = 0.024–0.333, *P* < 0.001), surgery (OR = 32.053, 95% CI = 0.007–0.137, *P* < 0.001), and chemotherapy (OR = 6.174, 95% CI = 0.038–0.688, *P*=0.014) were the independent risk factors for *E. coli* BSI of pancreatic cancer patients. *E. coli* resistant to carbapenems was rare; they were susceptible to cephamycin and piperacillin/tazobactam. The 90-day mortality rate of the infected group was significantly higher than the control group (41.9% versus 8.6%, *P* < 0.001).

**Conclusions:**

Hospitalization days ≥7 days, surgery, and chemotherapy are the independent risk factors for *E. coli* BSI of pancreatic cancer patients, which allows us to identify patients at potential risk and perform preventive treatment in time.

## 1. Introduction

Bloodstream infection (BSI) has a high mortality rate and is costly to treat; it is the most challenging type of infection to control and has a significant impact on the prognosis of patients [1]. Due to the improper use of antibiotics, pathogenic bacteria's resistance to commonly used antibacterial drugs is increasing [2], coupled with the adverse reactions caused by the abuse of antibiotics [3], which has made the treatment of BSI difficult.

In patients with malignant tumors, due to integrity damage (e.g., surgery, trauma, and indwelling catheters) and some medical sources (e.g., antibiotic use, radiotherapy, and chemotherapy) or nonmedical factors (such as advanced age and poor nutritional status), the body's immunity is reduced or dysfunctions, and thus can easily induce various infections [4].

There are differences in the distribution and drug resistance of pathogens in BSI in different cancer patients [5]. We analyzed the BSI data of Tianjin Medical University Cancer Institute and Hospital (TMUCIH) for nine years and found that the most common pathogen of BSI in cancer patients was *Escherichia coli* (*E. coli*), and pancreatic cancer patients rank as the first of *E. coli* BSI.

*E. coli* is the leading cause of BSI involving Gram-negative bacteria [6]. The last 20 years have witnessed a striking increase of BSI caused by antibiotic-resistant isolates of *E. coli* [7, 8]. Studies have shown that failure to provide timely and effective antibacterial therapy for BSI caused by extended-spectrum-lacta-mases (ESBL)-producing *E. coli* is associated with increased mortality [7, 9].

Pancreatic cancer is a malignant tumor of the digestive tract with high malignancy and difficulty in diagnosis and treatment [10, 11]. Postoperative adjuvant chemotherapy such as radiotherapy and chemotherapy can improve the survival rate; however, the 5-year survival rate is only around 5%, which is still one of the worst prognostic malignancies [10, 12]. Early diagnosis and treatment are key to improve the prognosis of pancreatic cancer [13].

There are 2,600 beds in TMUCIH, more than 80 beds in the hepatobiliary and gastrointestinal oncology departments, and only 30 beds in the pancreatic oncology department. However, the proportion of *E. coli* BSI was the highest, of which the cause remained to be determined. Further analysis of the risk factors can assist in the diagnosis and treatment of pancreatic cancer patients with BSI. For various reasons, patients with solid tumors are particularly predisposed to develop BSI; however, little information is currently available, let alone pancreatic cancer. Therefore, our data are precious and can provide valuable experience.

## 2. Materials and Methods

### 2.1. Patients and Study Design

Our study analyzed 2386 blood culture-positive pathogens of cancer patients from January 2011 to December 2019 in TMUCIH (exclude duplicate isolates isolated from the same patient). The first ranked pathogen was *E. coli* (697 cases). The primary cancer patients with *E. coli* BSI were pancreatic cancer patients (100 cases). 31 pancreatic cancer patients with *E. coli* BSI were randomly selected and defined as the infected group, and 93 pancreatic cancer patients without BSI (no blood culture was drawn during hospitalization and have no episodes of Gram-negative bacteremia), sex, age, and admission time matched with infected group, were collected as the control group. All cancer types in this study were confirmed by pathological diagnosis. The diagnosis of pancreatic cancer was made prior to hospitalization with *E. coli* BSI or made during the same hospitalization but prior to the onset of *E. coli* BSI.

The clinical data of the infected and control groups were retrospectively analyzed by a case-control study. The clinical data, including gender, age, the number of hospitalization days, clinical stage, the number of admissions, surgery, intraoperative bleeding, chemotherapy, radiotherapy, the type of antibiotics used, distant organ metastasis of cancer, invasive operation (central venous catheter, drainage tube, or urine tube), merger with other parts of infection, stayed in ICU, blood transfusion, combined with diabetes, glutamine transfusion, white blood cells <4.0 × 10^9^/L, neutropenia (neutrophil<1.5 × 10^9^/L), albumin<40.0 g/L, and prealbumin < 0.2 g/L, were collected. All of the above factors occurred during the admission and within 30 days before the onset of *E. coli* BSI. Clinical staging of pancreatic cancer was performed according to the American Cancer Society Tumor Staging Manual, 8th edition. TNM staging was performed first, with stages I and II being early and stages III and IV being late. The drainage tube was placed during the hospitalization but prior to *E. coli* BSI. Stayed in ICU was only this hospitalization before *E. coli* BSI. The number of admissions was restricted to admissions since the diagnosis of pancreatic cancer prior to *E. coli* BSI. The laboratory values were baseline values, which were the first value of the admission. The 90-day mortality rate (referred to the ratio of death within 90 days after the first positive blood culture) of the infected group and the control group was compared.

### 2.2. Ethics Statement

This study was approved by the Research Ethics Committee of TMUCIH. Informed consent was obtained from all the patients according to the regulation of the Institutional Review Boards of TMUCIH in agreement with the Declaration of Helsinki.

### 2.3. Definition

Hospital-acquired BSI was defined as the first positive culture obtained at 48 h after hospital admission or 48 h of discharge, along with clinical signs of active infection. When there was an unexplained fever and suspected to be a BSI, doctors would send at least one set of blood cultures and promptly sent for inspection. Blood samples (8–10 mL) were collected and auto-cultured by BACTEC 9050, 9120, or FX400 (Becton Dickinson, Franklin Lakes, NJ, USA) for 5 days. Positive samples were subcultured on blood agar (Jin Zhang Ke Ji, Tianjin, China) at 35°C for 24–48 hours depending on the results of Gram staining. Species identification and bacterial susceptibility tests were performed on a VITEK2 Compact automatic microbiological analysis system (bio-Merieux SA, Marcy l'Etoile, France); all coincidence rates were above 95%.

### 2.4. Quality Control

The quality control isolates were *Enterobacter cloacae* ATCC700323, *E. coli* ATCC25922, and *Pseudomonas aeruginosa* ATCC27853.

### 2.5. Statistical Analysis

Bacterial resistance analysis, pathogen, and tumor-type distribution were performed by Whonet 5.6 software. SPSS 17.0 was adopted to perform univariate and multivariate analyses. Data of categorical variables were compared by Fisher's exact test; *P* < 0.1 was defined as a potential risk factor. The potential risk factors were included in the multivariate logistic regression model to analyze the independent risk factors; *P* < 0.05 indicates statistical significance; all tests were two-tailed.

## 3. Results

### 3.1. Distribution of Pathogenic Bacteria in BSI of Cancer Patients

In 2011–2019, 2386 isolates of pathogens were detected in blood culture-positive specimens. The main pathogens causing BSI were *E. coli* (697,29.21%), coagulase-negative staphylococci (387, 16.22%), *Klebsiella pneumoniae* (318, 13.33%), *Staphylococcus aureus* (139, 5.83%), *Pseudomonas aeruginosa* (94, 3.94%), *Enterobacter cloacae* (88, 3.69%), *Enterococcus faecalis* (77, 3.23%), *Enterococcus* faecium (70, 2.93%), *Candida* albicans (46, 1.93%), and *Klebsiella* oxytoca (40, 1.68%). The distribution of the pathogens is shown in Table 1.

### 3.2. Tumor-Type Distribution of Cancer Patients Complicated with *E. coli* BSI

The BSI caused by *E. coli* was distributed in multiple cancers. The main tumor types with *E. coli* BSI were pancreatic cancer (100 cases, 14.35%), gastric cancer (64, 9.18%), lymphoma (55, 7.89%), cholangiocarcinoma (54, 7.75%), lung cancer (40, 5.74%), breast cancer (36, 5.16%), colon cancer (31, 4.45%), cervical cancer (28, 4.02%), rectal cancer (27, 3.87%), and liver cancer (27, 3.87%). The distribution of tumor types is shown in Table 2.

### 3.3. Analysis of Risk Factors for *E. coli* BSI of Patients with Pancreatic Cancer

The clinical data of 31 infected patients and 93 controls were analyzed, including gender, age, days of hospitalization, clinical stage, number of admissions, surgery, intraoperative bleeding, chemotherapy, radiotherapy, and other characteristics. The results showed that hospitalization days ≥7 days, number of admissions ≥2 times, surgery, chemotherapy, the type of antibiotics used ≥2 species, albumin <40.0 g/L, and prealbumin <0.2 g/L were the potential risk factors for pancreatic cancer patients with *E. coli* BSI (*P* < 0.1); gender, age, clinical stage, intraoperative bleeding, radiotherapy, and other characteristics were not the potential risk factors for pancreatic cancer patients with *E. coli* BSI (*P* < 0.1). The 90-day mortality rate of the infected group was significantly higher than that of the control group (41.9% versus 8.6%, *P* < 0.001), see Table 3.

### 3.4. Multivariate Logistic Regression Analysis of *E. coli* BSI in Patients with Pancreatic Cancer

Incorporate the potential risk factors into the multivariate logistic regression model. The results showed that hospitalization days ≥7 days (OR = 11.196, 95% CI = 0.024–0.333, *P* < 0.001), surgery(OR = 32.053, 95% CI = 0.007–0.137, *P* < 0.001), and chemotherapy (OR = 6.174, 95% CI = 0.038–0.688, *P*=0.014) were the independent risk factors for *E. coli* BSI in patients with pancreatic cancer (see Table 4).

### 3.5. Analysis of Drug Resistance of Pancreatic Cancer Patients Complicated with *E. coli* BSI

There was no significant difference in the resistance rates of antibiotics between *E. coli* BSI of pancreatic cancer and nonpancreatic patients. The ratio of *E. coli* producing extended-spectrum *ß*-lactamase was 49.0 and 48.1, respectively. *E. coli* resistant to carbapenems were rare; they were susceptible to cephamycin and piperacillin/tazobactam. The resistance data are shown in Table 5.

## 4. Discussion

BSI refers to the invasion of various pathogenic microorganisms (bacteria or fungi) into the bloodstream and is a serious systemic infectious disease [14]. The proportion of Gram-positive BSI has increased in the last 20 years, but Gram-negative BSI still accounts for about 50%, and *E. coli* is the leading cause [15, 16]. Patients with malignant tumors often require surgery, high-dose chemoradiotherapy, antibiotics, and various invasive procedures; the malignancy itself tends to increase the chance of *E. coli* infection [17]. Blood culture is the gold standard for the diagnosis of *E. coli* BSI [18], but its diagnosis takes a long time, which will delay clinical and timely treatment. Besides, the distribution of pathogens and the risk factors for BSI are different due to different diseases and tumor types [19]. Pancreatic cancer has a high degree of malignancy, a low surgical resection rate, and a poor prognosis [20]. The occurrence of BSI may further increase the difficulty of treatment. Therefore, clinical analysis of the risk factors for *E. coli* BSI in patients with pancreatic cancer is essential for preventing the potentially high-risk populations and the timely, effective treatment of patients.

We analyzed the baseline characteristics in the infected and control groups that may be related to infection [21–23], including gender, age, days of hospitalization, clinical stage, number of admissions, surgery, intraoperative bleeding, chemotherapy, radiotherapy, and other characteristics. Our study revealed that hospitalization days ≥7 days, number of admissions ≥2 times, surgery, chemotherapy, the type of antibiotics used ≥2 species, albumin <40.0 g/L, and prealbumin <0.2 g/L were associated with *E. coli* BSI of pancreatic cancer patients; gender, age, clinical stage, intraoperative bleeding, radiotherapy, and other characteristics were not the potential risk factors for pancreatic cancer patients with *E. coli* BSI (*P* ≥ 0.1). Multivariate logistic regression analysis showed that only hospitalization days ≥7 days, surgery, and chemotherapy were the independent risk factors for *E. coli* BSI. Pancreatic cancer patients have a long-term adjuvant and neoadjuvant treatment, radiation therapy, combined with various serious underlying diseases and multiple surgical treatments leading to prolonged hospital stay [24]; this gradually reduces the immune function, thus increasing the chance of *E. coli* BSI. A study has also shown that patients with more extended hospital stays are prone to BSI [25]. Therefore, doctors and nurses in the hospital should strictly carry out the aseptic operation, i.e., regularly disinfect the medical equipment and related wards and departments, to reduce the incidence of BSI.

Surgery is a kind of trauma to the human body, which will cause the patient's resistance to decline, causing BSI [26]. Besides, with the rapid development and popularization of medical technology, various implant operations such as drainage tubes, catheters, central venous catheters, etc., play not only a therapeutic role but also pose a risk to BSI; therefore, implantation was also an important risk factor [27]. Our data showed that surgery was an independent risk factor for *E. coli* BSI of pancreatic cancer patients.

In the results of this study, 67.7% of pancreatic cancer patients with *E. coli* BSI received chemotherapy (adjuvant or/and neoadjuvant treatment). Neoadjuvant treatment helps kill cancer cells and reduce the tumor implantation caused by surgery. Adjuvant therapy can effectively improve the treatment effect and reduce recurrence and metastasis. Adjuvant chemotherapy with gemcitabine is a standard care for resected pancreatic cancer [28], not only delayed recurrence but also improved survival compared with surgery alone [29]. However, while killing cancer cells, chemotherapy also kills normal immune cells. For instance, the dose-limiting toxicity of gemcitabine is myelosuppression, leading to decreased neutrophils and platelets, which reduces the body's immunity. Simultaneously, chemotherapeutic drugs such as doxorubicin can easily damage the intima of the blood vessels [30], leading to partial venous catheterization blockage, causing BSI. Therefore, chemotherapy was an independent risk factor for *E. coli* BSI of pancreatic cancer patients. Cancer patients often have infections in other sites, and different types of antibiotics are often used for different infection sites [31]. We found that using two or more antibiotic types was a potential risk factor for *E. coli* BSI.

Neutrophils are the most abundant white blood cells, accounting for 50%–70% of the total white blood cells; they are the first barrier of the body's defense. When inflammation occurs, neutrophils will penetrate the vascular endothelium, and then enter the site of inflammation to exert a bactericidal effect. Patients with neutropenia may be prone to bloodstream infections. However, we found that neutropenia is not directly related to *E. coli* BSI, which may be due to patients with pancreatic cancer suffering from chemoradiotherapy, which inhibits bone marrow function [24]. Meanwhile, they received leukocyte ascending therapy, resulting in a normal number of white blood cells and neutrophils, but they usually failed to exert anti-inflammatory effects.

Albumin and prealbumin can be used as monitoring indicators of the nutrients in the body. When the nutritional status of the patients is poor, the immune function of the body decreases, and albumin and prealbumin decrease accordingly [32]. In our analysis, albumin <40.0 g/L and prealbumin <0.2 g/L were the potential risk factors, indicating that patients with pancreatic cancer were prone to *E. coli* BSI when their nutritional status is low.

In our study, the proportion of ESBL produced by *E. coli* was about 50%, which was lower than that reported in other studies [33]. The ceftriaxone resistance rate was significantly higher than that of ceftazidime, which may be related to the main cefotaxime (CTX) type of ESBL in this region. Carbapenems, cephamycin, and piperacillin/tazobactam can be used as the first choice for empirical use, but doctors should adjust the drug according to the drug susceptibility results of clinical microbiology to reduce the occurrence of superior and restricted antibiotic resistance.

However, this study also has some limitations. It is a single-center study, which can be combined for pancreatic cancer in North China, and the number of cases was small. The diagnosis and treatment of pancreatic cancer patients with *E. coli* BSI need further multi-center, large sample size research to promote its application.

In summary, pancreatic cancer patients with *E. coli* BSI exhibited higher mortality than control groups; patients with suspected *E. coli* BSI should be promptly drawn for blood culture. Hospitalization days ≥7 days, surgery, and chemotherapy are the independent risk factors for *E. coli* BSI; this allows us to identify patients at potential risk and perform preventive treatment in a short period. Early use of medication, while timely adjustment based on clinical drug sensitivity results, will also help reduce patients' mortality.

## Figures and Tables

**Table 1:** The ratio of pathogens in cancer patients complicated with BSI.

Pathogen	Number of isolates	Composition ratio (%)
*Escherichia coli*	697	29.21
Coagulase-negative staphylococci	387	16.22
*Klebsiella pneumoniae*	318	13.33
*Staphylococcus aureus*	139	5.83
*Pseudomonas aeruginosa*	94	3.94
*Enterobacter cloacae*	88	3.69
*Enterococcus faecalis*	77	3.23
*Enterococcus faecium*	70	2.93
*Candida albicans*	46	1.93
*Klebsiella oxytoca*	40	1.68
*Acinetobacter baumannii*	26	1.09
*Stenotrophomonas maltophilia*	25	1.05
*Streptococcus anginosus*	25	1.05
*Serratia marcescens*	21	0.88
*Streptococcus mitis*	19	0.80
*Enterobacter aerogenes*	17	0.71
*Proteus mirabilis*	17	0.71
*Streptococcus intermedius*	14	0.59
*Streptococcus constellatus*	13	0.54
*Citrobacter freundii*	13	0.54
*Candida parapsilosis*	12	0.50
*Enterococcus gallinarum*	12	0.50
*Streptococcus pneumoniae*	12	0.50
*Burkholderia cepacia*	10	0.42
*Salmonella* sp.	30	1.26
*Candida glabrata*	9	0.38
*Aeromonas hydrophila*	9	0.38
*Enterococcus avium*	9	0.38
*Morganella morganii*	8	0.34
*Candida tropicalis*	8	0.34
*Streptococcus pyogenes*	6	0.25
*Acinetobacter lwoffii*	5	0.21
*Candida famata*	5	0.21
*Pantoea agglomerans*	5	0.21
*Acinetobacter junii*	5	0.21
*Streptococcus* sanguinis	5	0.21
Other bacteria	90	3.77
Total	2386	100.00

**Table 2:** Tumor-type distribution of cancer patients complicated with *E. coli* BSI.

Tumor type	Cases	Composition ratio (%)
Pancreatic cancer	100	14.35
Gastric cancer	64	9.18
Lymphoma	55	7.89
Cholangiocarcinoma	54	7.75
Lung cancer	40	5.74
Breast cancer	36	5.16
Colon cancer	31	4.45
Cervical cancer	28	4.02
Rectal cancer	27	3.87
Liver cancer	27	3.87
Duodenal cancer	26	3.73
Ovarian cancer	26	3.73
Gallbladder cancer	16	2.30
Sarcoma	14	2.01
Bladder cancer	14	2.01
Endometrial cancer	13	1.87
Prostate cancer	12	1.72
Leukemia	12	1.72
Benign tumor	11	1.58
Kidney cancer	8	1.15
Head and neck cancer	7	1.00
Esophageal cancer	7	1.00
Metastatic cancer	5	0.72
High-grade squamous intraepithelial lesion of the cervix	5	0.72
Inflammation	4	0.57
Vulvar cancer	4	0.57
Neuroblastoma	4	0.57
Benign prostatic hyperplasia	4	0.57
Meningioma	4	0.57
Plasmacytoma	4	0.57
Stromal tumor	4	0.57
Penile cancer	3	0.43
Pancreatic neuroendocrine tumor	3	0.43
Serous cystadenoma of the pancreas	3	0.43
Peritoneal cancer	3	0.43
Multiple myeloma	3	0.43
Vaginal cancer	2	0.29
Glioma	2	0.29
Melanoma	2	0.29
Other malignant tumors	10	1.43
Total	697	100.00

**Table 3:** Analysis of risk factors for *E. coli* BSI in patients with pancreatic cancer.

Risk factor	Category	Infected group (*n* = 31)		Control group (*n* = 93)		*p* value
		Number of cases	Composition ratio (%)	Number of cases	Composition ratio (%)	
Gender	Male	18	58.1	51	54.8	0.836
	Female	13	41.9	42	45.2	

Age (years)	≥60	15	48.3	56	60.2	0.297
	<60	16	51.7	37	39.8	

Hospitalization days	≥7	17	54.8	11	11.8	<0.001
	<7	14	45.2	82	88.2	

Clinical stage	Late	27	87.1	74	79.6	0.432
	Early	4	12.9	19	20.4	

Number of admissions (times)	≥2	30	96.8	79	84.9	0.067
	<2	1	3.2	14	15.1	

Surgery	Yes	18	58.1	6	6.5	<0.001
	No	13	41.9	87	93.5	

Intraoperative bleeding	Yes	6	19.4	22	23.7	0.805
	No	25	80.6	71	76.3	

Chemotherapy	Yes	21	67.7	46	49.5	0.097
	No	10	32.3	47	50.5	

Radiotherapy	Yes	1	3.3	0	0.0	0.250
	No	30	96.7	93	100.0	

Type of antibiotics used (species)	≥2	9	29.0	12	12.9	0.052
	<2	22	71.0	81	87.1	

Distant organ metastasis of cancer	Yes	16	51.6	35	37.6	0.208
	No	15	48.4	58	62.4	

Central venous catheter	Yes	1	3.3	0	0.0	0.250
	No	30	96.7	93	100.0	

Drainage tube	Yes	12	38.7	32	34.4	0.670
	No	19	61.3	61	65.6	

Merger with other parts of infection	Yes	6	19.4	16	17.2	0.790
	No	25	80.6	77	82.8	

Stayed in ICU	Yes	5	16.1	8	8.6	0.308
	No	26	83.9	85	91.4	

Blood transfusion	Yes	5	16.1	18	19.4	0.795
	No	26	83.9	75	80.6	

Combined with diabetes	Yes	5	16.1	25	26.9	0.333
	No	26	83.9	68	73.1	

Glutamine transfusion	Yes	9	29.0	37	39.8	0.391
	No	22	71.0	56	60.2	

White blood cells (/L)	<3.5 × 109	2	6.5	9	9.7	0.729
	≥3.5 × 109	29	93.5	84	90.3	

Neutropenia	Yes	1	3.2	4	4.3	1.000
	No	30	96.8	89	95.7	

Albumin (g/L)	<40.0	24	77.4	48	51.6	0.012
	≥40.0	7	22.6	45	48.4	

Prealbumin (g/L)	<0.2	28	90.3	65	69.9	0.030
	≥0.2	3	9.7	28	30.1	

Death	Yes	13	41.9	8	8.6	<0.001
	No	18	58.1	85	91.4	

**Table 4:** Multivariate logistic regression analysis of *E. coli* BSI in patients with pancreatic cancer.

Risk factor	B	Wald *χ*^2^	OR = Exp (B)	*P* value	95% CI
Hospitalization days ≥7 days	2.416	12.921	11.196	<0.001	0.024–0.333
Number of admissions ≥2 times	0.49	0.094	1.633	0.76	0.026–14.174
Surgery	3.467	21.155	32.053	<0.001	0.007–0.137
Type of antibiotics used ≥2 species	0.771	1.004	2.163	0.316	0.102–2.091
Chemotherapy	1.82	6.084	6.174	0.014	0.038–0.688
Albumin <40.0 g/L	0.834	1.338	2.301	0.247	0.106–1.784
Prealbumin <0.2 g/L	1.198	1.701	3.314	0.192	0.05–1.827

**Table 5:** Analysis of drug resistance of *E. coli* BSI in pancreatic cancer and nonpancreatic patients.

Antibiotic	Pancreatic cancer (*n* = 100)	Nonpancreatic cancer (*n* = 597)	*P* value
ESBL	49.0	48.1	0.898
Ampicillin	82.0	80.6	0.799
Piperacillin	60.0	61.6	0.817
Ampicillin/sulbactam	52.0	45.1	0.329
Piperacillin/tazobactam	3.0	2.7	0.766
Cefazolin	55.0	51.4	0.61
Cefuroxime	49.0	50.3	0.854
Ceftazidime	23.0	20.8	0.707
Ceftriaxone	51.0	49.7	0.854
Cefepime	16.0	12.7	0.506
Cefotetan	2.0	2.2	0.693
Aztreonam	30.0	30.2	0.975
Imipenem	0.0	0.2	0.803
Meropenem	0.0	0.2	0.803
Amikacin	3.0	0.8	0.268
Gentamicin	39.0	48.7	0.167
Tobramycin	10.0	10.7	0.871
Ciprofloxacin	39.0	50.9	0.091
Levofloxacin	38.0	48.1	0.149
Sulfamethoxazole	57.0	60.1	0.656

## Data Availability

The data that support the findings of this study are available from the archives of the TMUCIH, but restrictions apply to the availability of these data, which were used under license for the current study, and so are not publicly available. Data are however available from the authors upon reasonable request to the corresponding author (Yueguo Li) and with the permission of the TMUCIH.
